# Supplementary figures and images for: TaTLP1 interacts with TaPR1 to contribute to wheat defense responses to leaf rust fungus
Source: PLoS Genet. 2020 Jul 13;16(7):e1008713. doi: 10.1371/journal.pgen.1008713 (PMC7357741; doi:10.1371/journal.pgen.1008713)

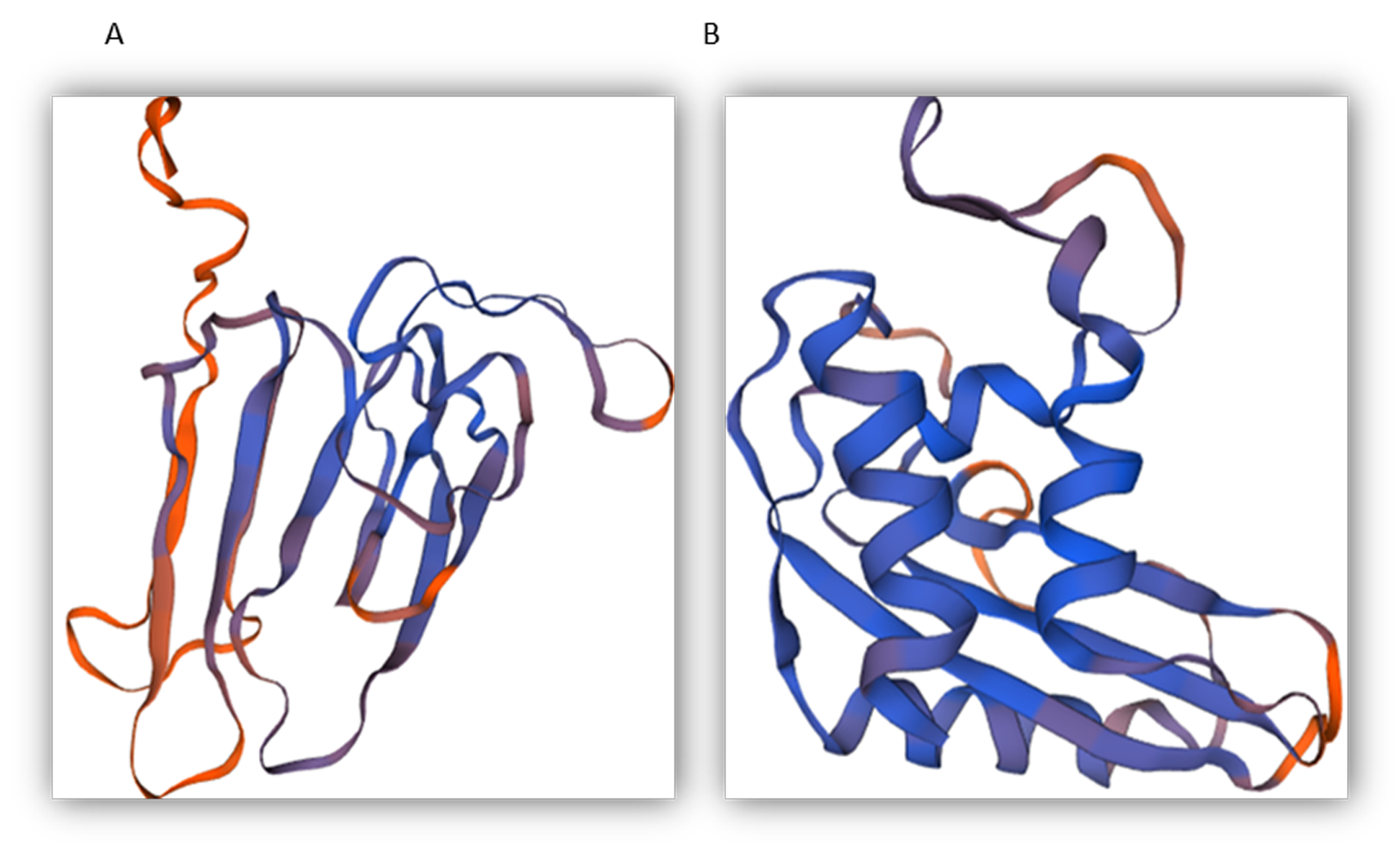

Supplement: S1 Fig — The structure of TaTLP1 (A) and TaPR1 (B) were shown as diagram and surface views. The N-terminal region, C-terminal region and ΔC113-164 are shown in these proteins structure of TaTLP1 and TaPR1. Online software SWISS-MODEL was used to predict these proteins the second structures. (TIF) [file pgen.1008713.s001.tif]

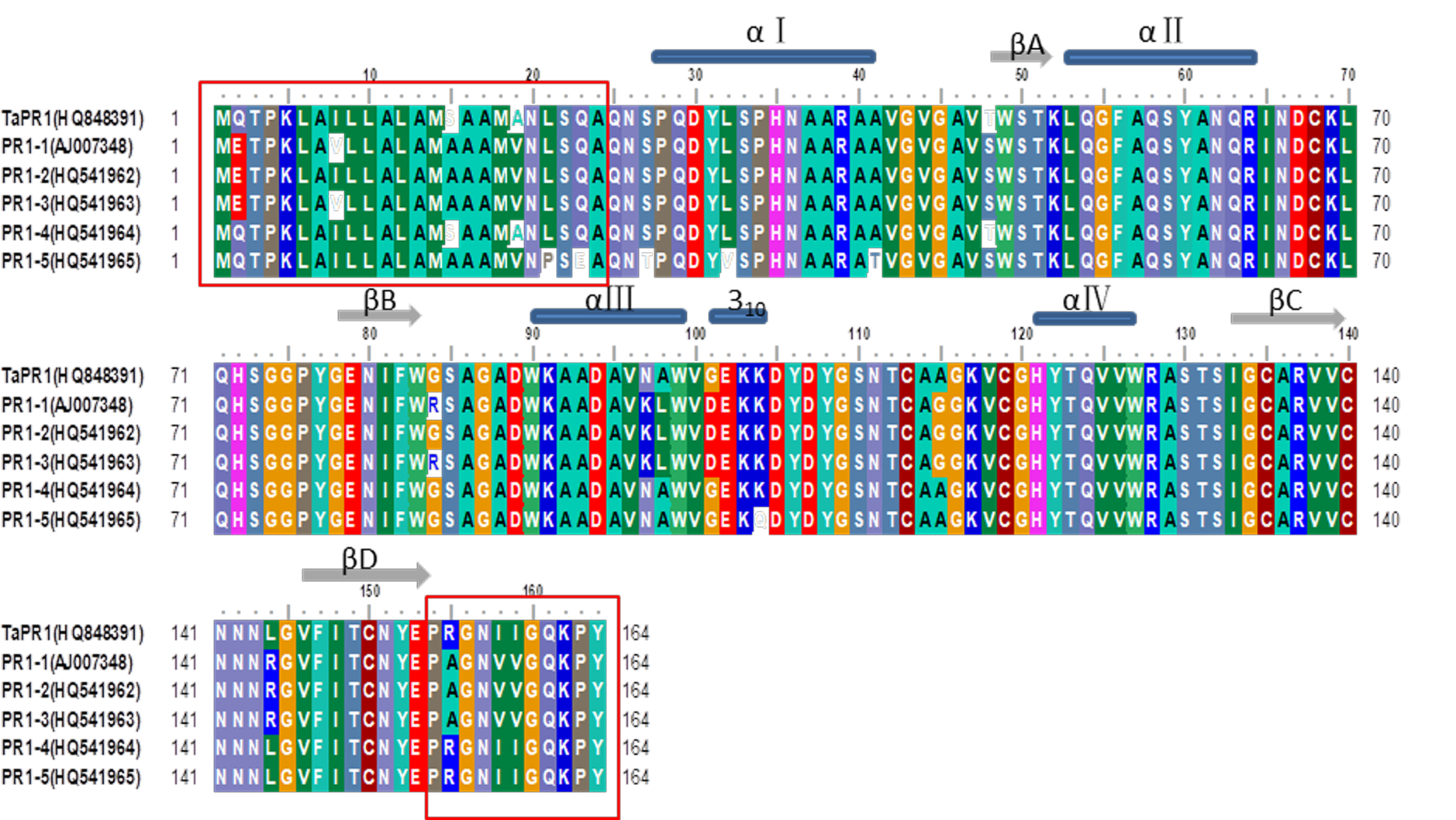

Supplement: S2 Fig — Sequence from NCBI (https://www.ncbi.nlm.nih.gov/). Gene ID: TaPR1 from our previous study, ID: HQ848391, PR1-1’s gene ID: AJ007348, PR1-2’s gene ID: HQ541962, PR1-3’s gene ID: HQ541963, PR1-4’s gene ID: HQ541964, PR1-5’s gene ID: HQ541965. TaPR1 is consistent with PR1-4 sequence. Red box indicated signal peptide and CAPE1. (TIF) [file pgen.1008713.s002.tif]

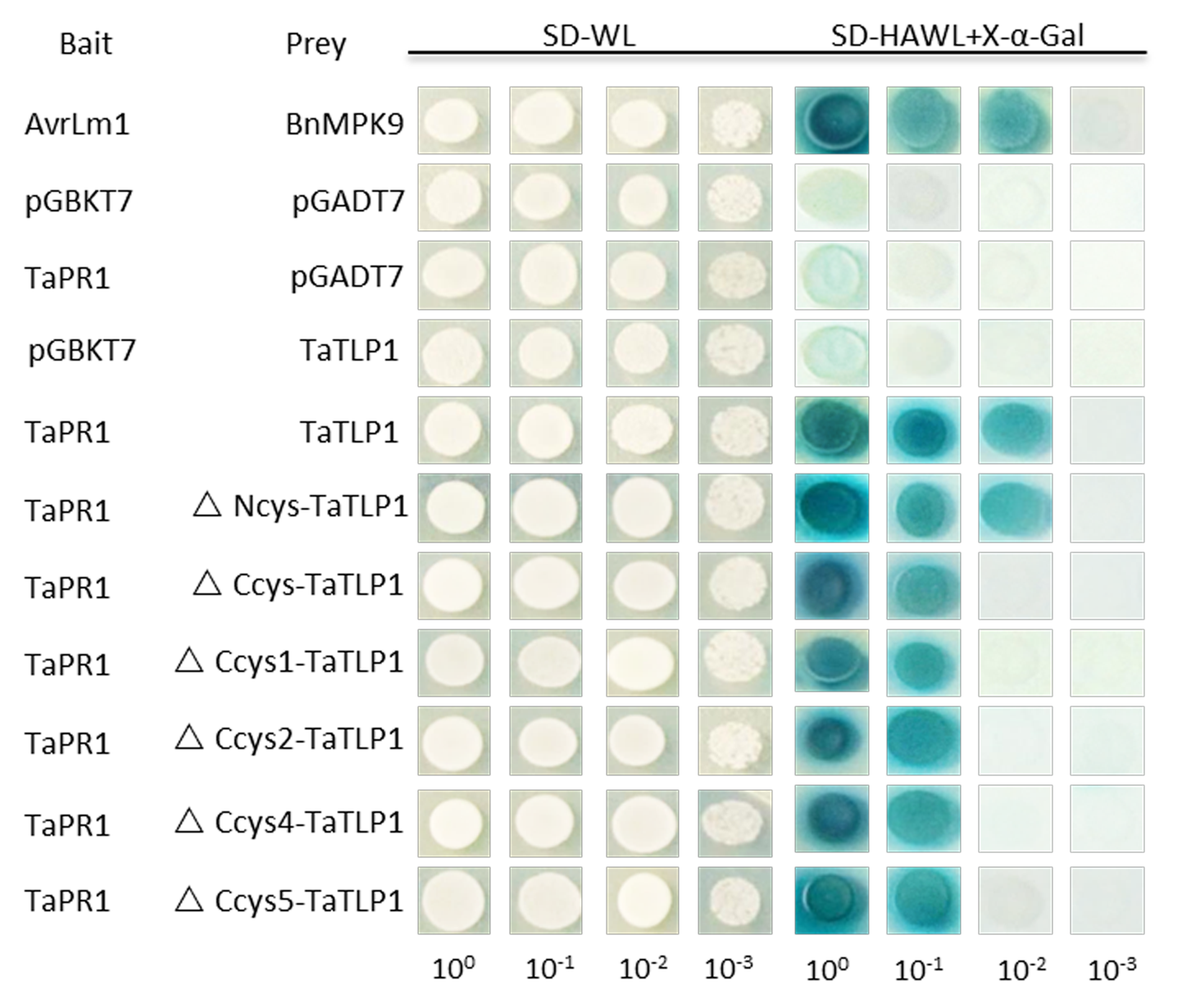

Supplement: S3 Fig — Two mutated TaTLP1 proteins with five cysteines to alanines at N-terminal region (ΔNcys-TaTLP1) and another five cysteines to alanines at C-terminal region (ΔCcys-TaTLP1), and four individual mutated cysteine at C-terminal (ΔCcys1-TaTLP1, ΔCcys2-TaTLP1, ΔCcys4-TaTLP1, ΔCcys5-TaTLP1) that were used for Y2H with TaPR1 respectively. All transformants are able to grow on synthetic dropout medium without leucine and tryptophan (SD-WL) medium. Yeast colonies that were able to grow on selective medium (SD medium without leucine, tryptophan, histidine, and adenine supplemented with X-α-Gal and AureobasidinA [SD-HAWL]) and displayed blue coloration confirmed the protein-protein interaction. Positive control is MAPK9-AvrLm1. TaPR1 and pGADT7, TaTLP1 and pGBKT7, pGADT7 and pGBKT7 are used as the negative controls. (TIF) [file pgen.1008713.s003.tif]

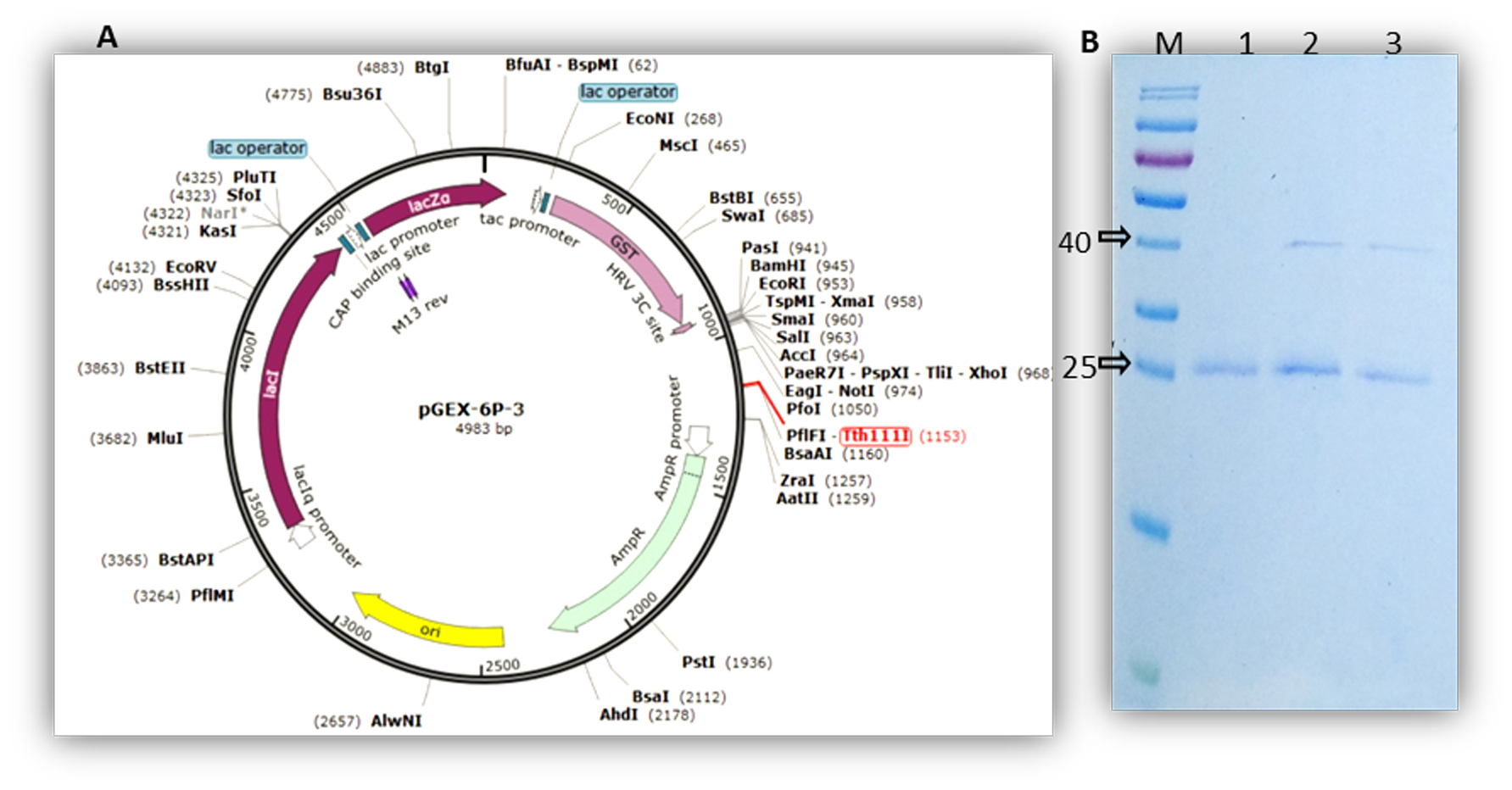

Supplement: S4 Fig — (A) Map of plasmid pGEX-6P-3. TaTLP1 and TaPR1 were cloned into the BamH I and EcoR I restriction sites of plasmid pGEX-6P-3 to generate the plasmid TaTLP1-GST, TaPR1-GST. The recombinant plasmid contains a GST tag. (B) Detection of purified proteins by SDS-PAGE. Lanes 1, 2 and 3 show pGEX-6P-3, TaTLP1-GST, TaPR1-GST expressed by E. coli Top10, respectively. The proteins were stained with Coomassie Brilliant Blue. M: Protein marker. The sites of restriction endonucleases are BamH I and EcoR I. The black arrow showing 18 kDa indicated the TaTLP1-GST and TaPR1-GST respectively; The white arrow showing 25 kDa indicated GST. (TIF) [file pgen.1008713.s004.tif]

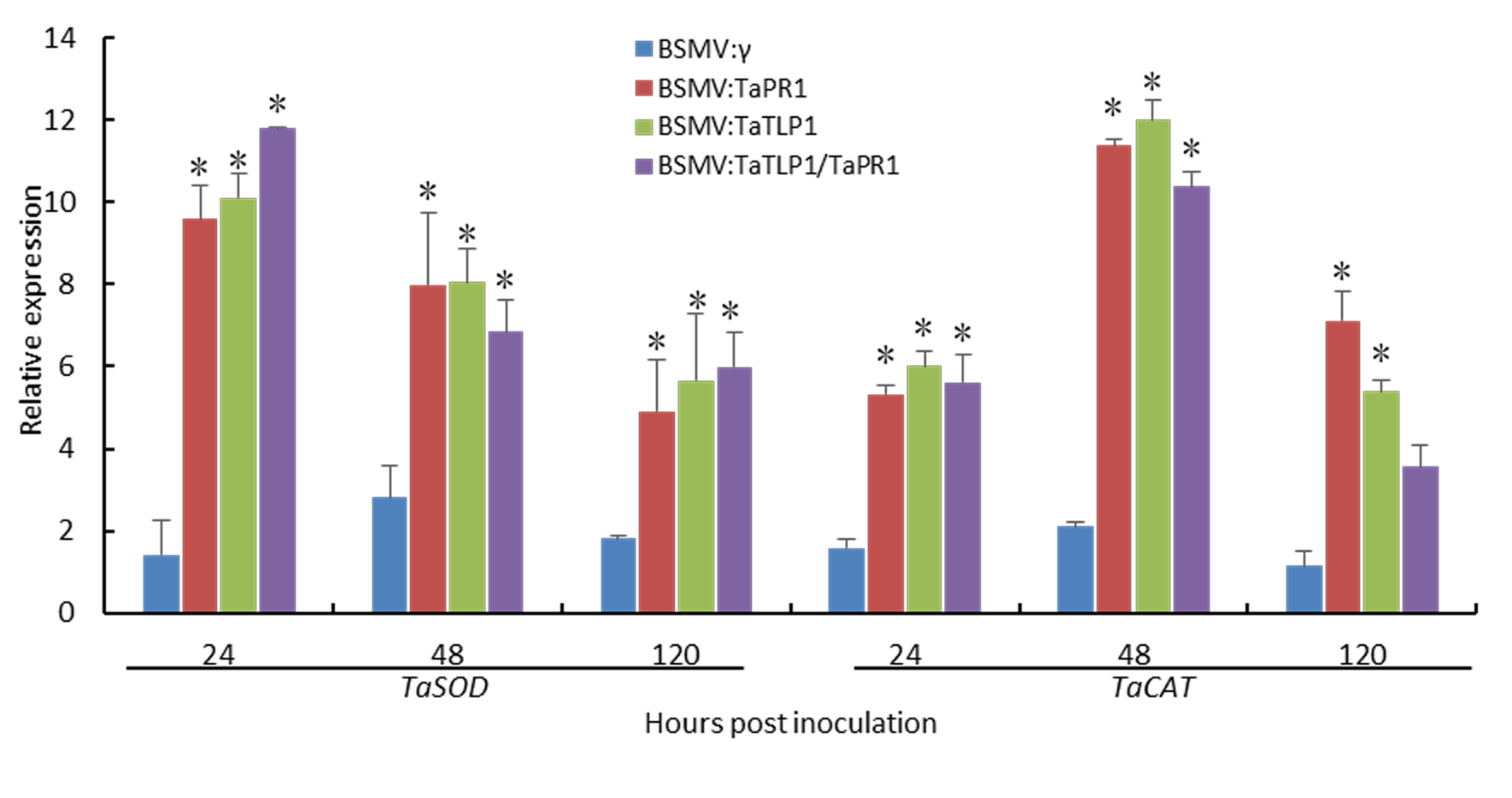

Supplement: S5 Fig — The y-axis indicates the amounts of TaSOD or TaCAT transcript normalized to the GAPDH gene. The x-axis indicates sampling times. The transcript levels of TaCAT and TaSOD in BSMV: γ were standardized as 1. Data are means ± standard errors (SE) of three independent experiments. Differences between that in knockdown plants and control plants were assessed using Student’s t-tests (* p < 0.05). (TIF) [file pgen.1008713.s005.tif]

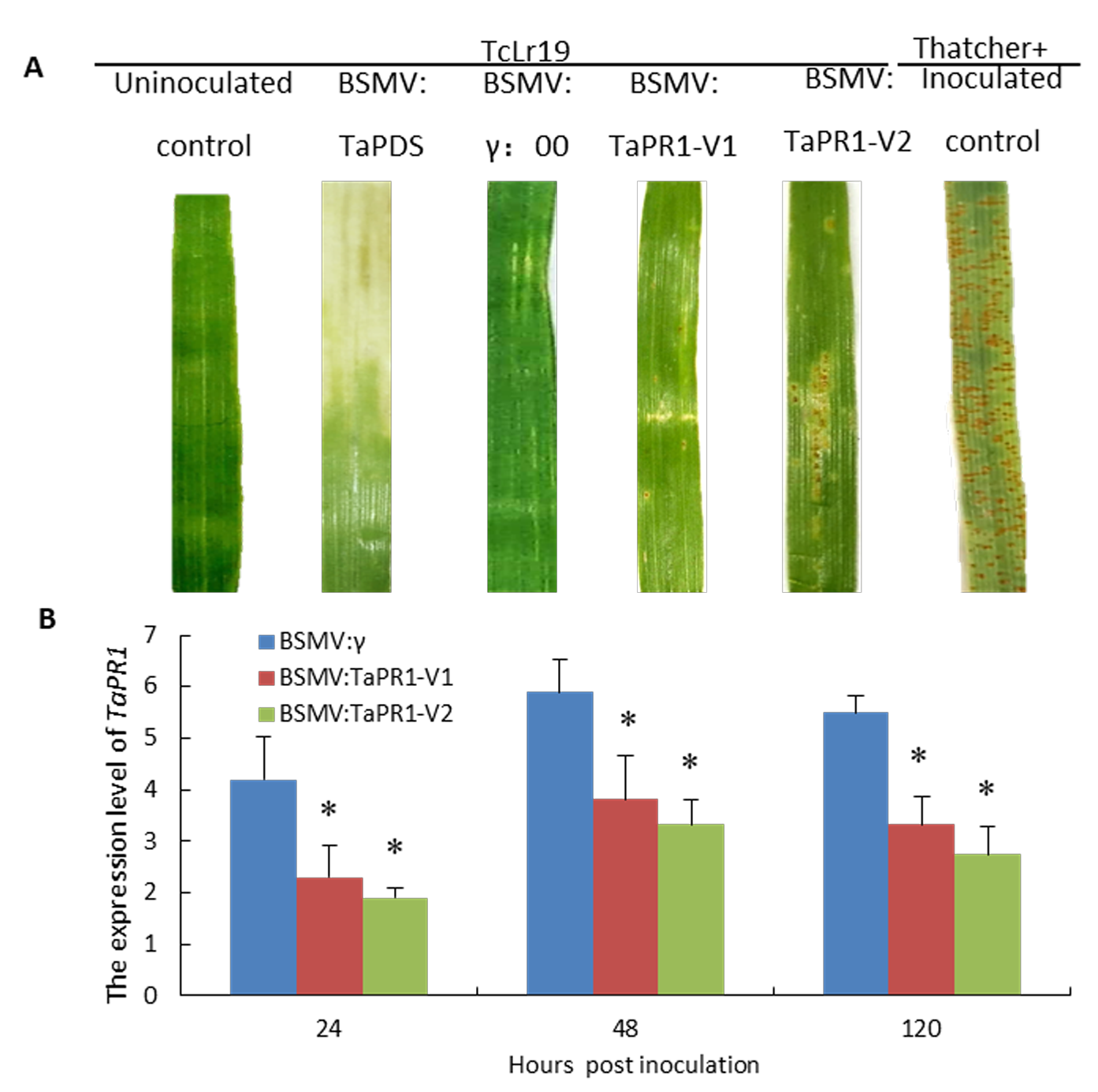

Supplement: S6 Fig — (A) Disease development in different silenced leaves after inoculation with leaf rust. Fourth leaves after infection with BSMV: TaPR1-V1, and BSMV: TaPR1-V2 at 14 dpi. BSMV: γ, BSMV: TaPDS, inoculated Thatcher and uninoculated leaves were used as negative and positive control, respectively. (B) Relative transcript levels of TaPR1 in knockdown plants at 24, 48 and 120 hpi. The y-axis indicates the amounts of TaPR1 transcript normalized to the GAPDH gene. The x-axis indicates sampling times. The transcript levels of TaPR1 in BSMV: γ was standardized as 1. Data are means ± standard errors (SE) of three independent experiments. Differences between that in knockdown plants and control plants were assessed using Student’s t-tests (* p < 0.05). (TIF) [file pgen.1008713.s006.tif]

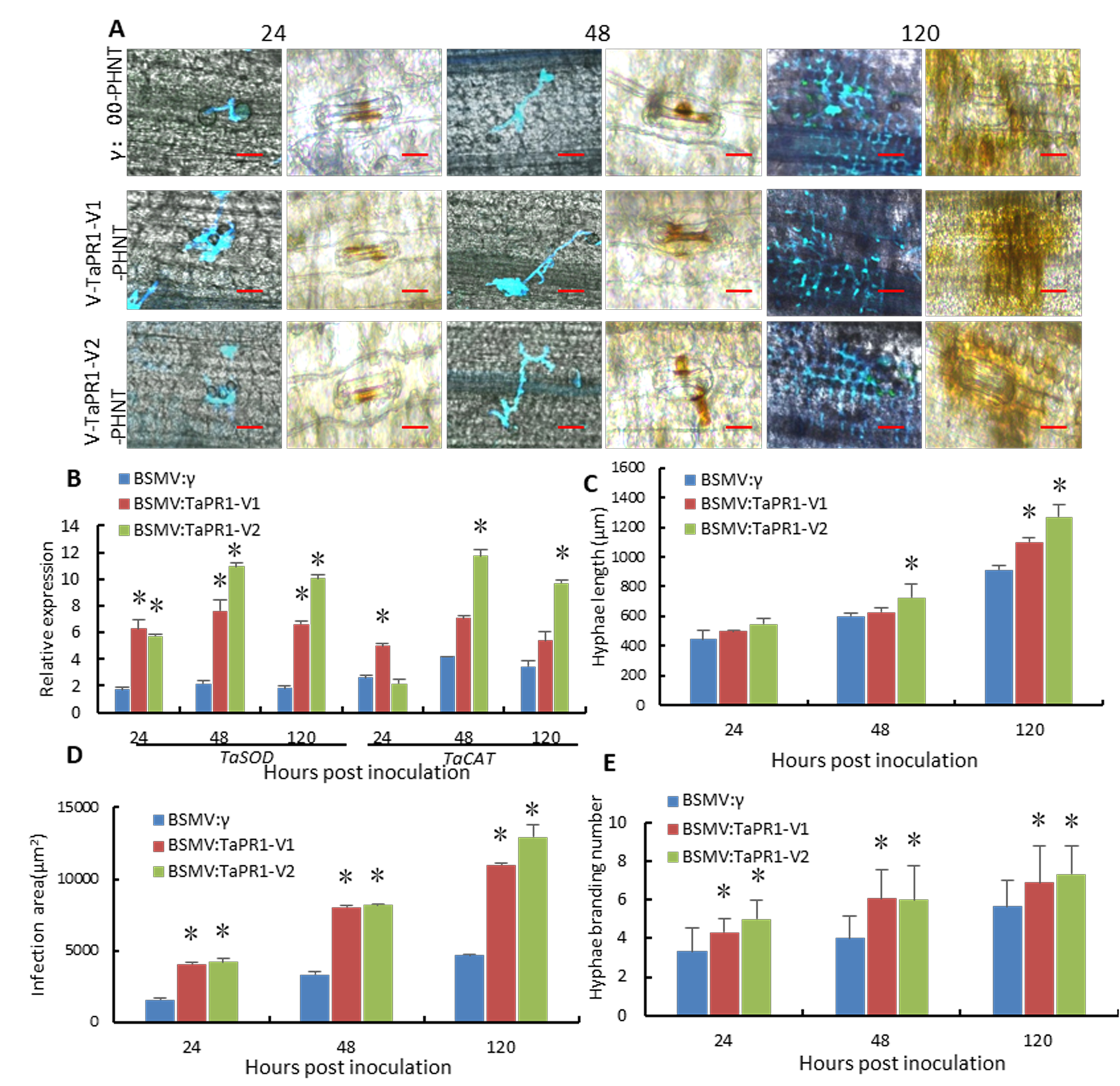

Supplement: S7 Fig — (A) Histological observation of hypha development and host cell death in wheat leaves BSMV-infected wheat leaves after inoculation with Pt at 24, 48 and 120 hpi. Scale bar, 50μm. H2O2 accumulation at infection sites was detected. Scale bar, 20 μm. (B) Detection of TaSOD and TaCAT expression in gene silencing plants. The y-axis indicates the amounts of TaSOD or TaCAT transcript normalized to the GAPDH gene. The x-axis indicates sampling times. The transcript levels of TaCAT and TaSOD in BSMV: γ were standardized as 1. Data are means ± standard errors (SE) of three independent experiments. Differences between that in knockdown plants and control plants were assessed using Student’s t-tests (* p < 0.05). (C, D, E) The fungal growth including the length of the hyphae, the area of infection, and the number of mycelial branches of Pt in wheat leaves inoculated with BSMV: γ, BSMV: TaPR1-V1 and BSMV: TaPR1-V2 at 24, 48, and 120 hpi was observed. The fungal growth level of BSMV: γ was standardized as 1. Data are means ± standard errors (SE) of three independent experiments. Differences were assessed using Student’s t-tests (* p < 0.05). (TIF) [file pgen.1008713.s007.tif]

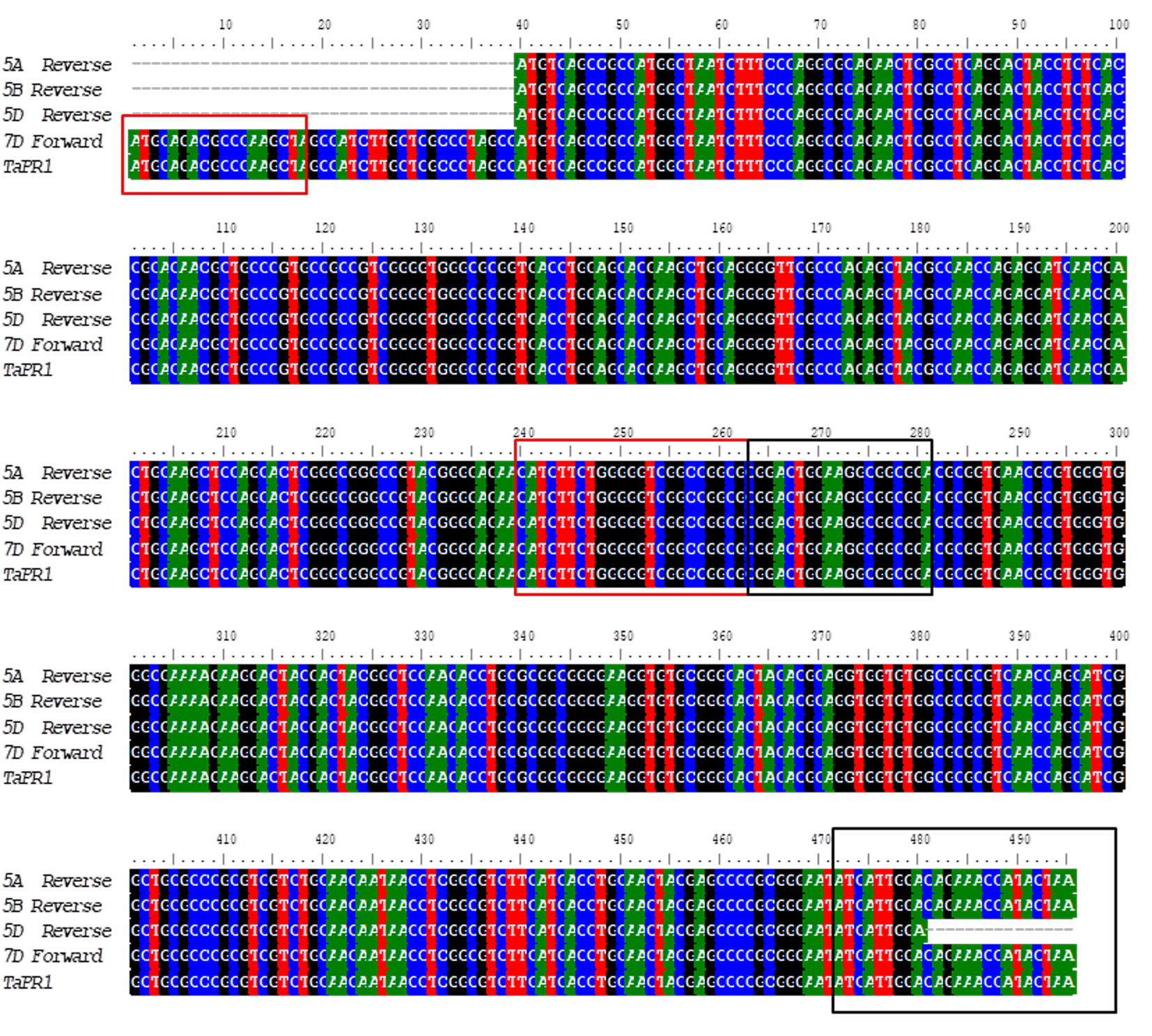

Supplement: S8 Fig — Multiple sequence alignment of the coding sequences for the four TaPR1 copies was performed by BioEdit software. The fragments for VIGS are indicated by red box (TaPR1-V1) and black box (TaPR1-V2), respectively. (TIF) [file pgen.1008713.s008.tif]

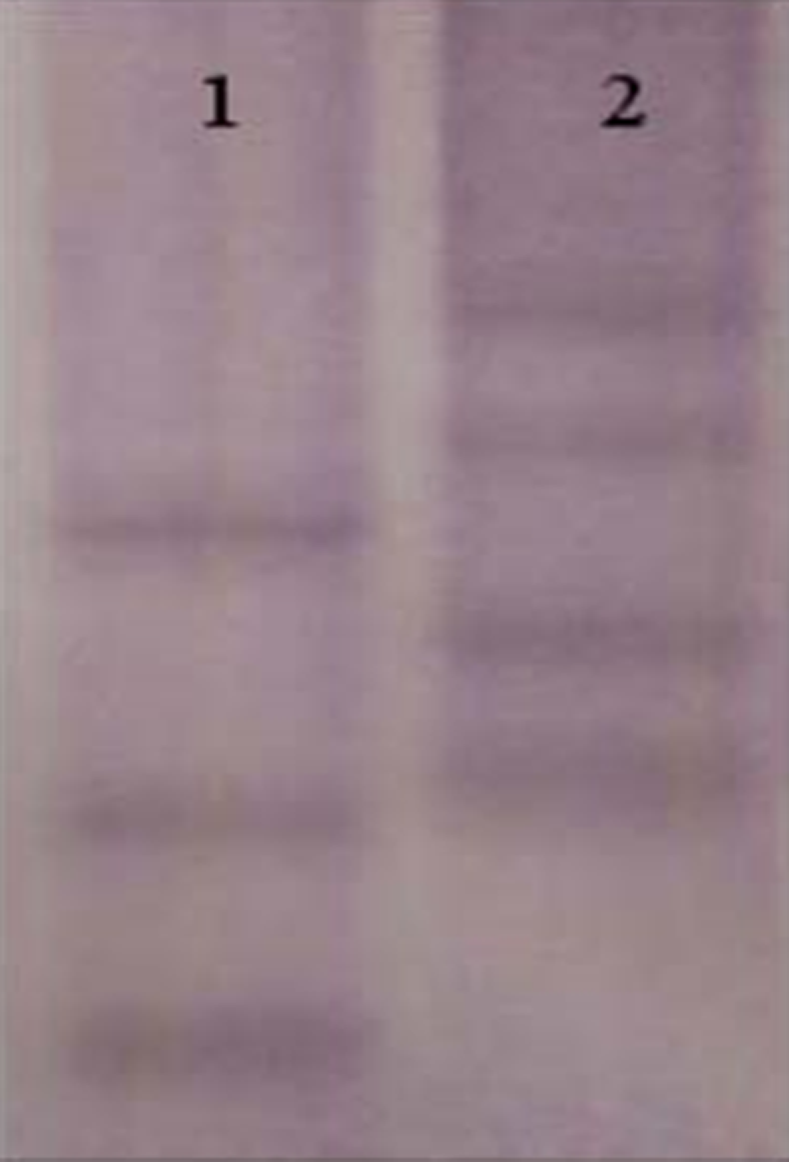

Supplement: S9 Fig — 1–2: TcLr19 DNA was digested with Hind Ⅲ、Sac Ⅰ respectively. (TIF) [file pgen.1008713.s009.tif]

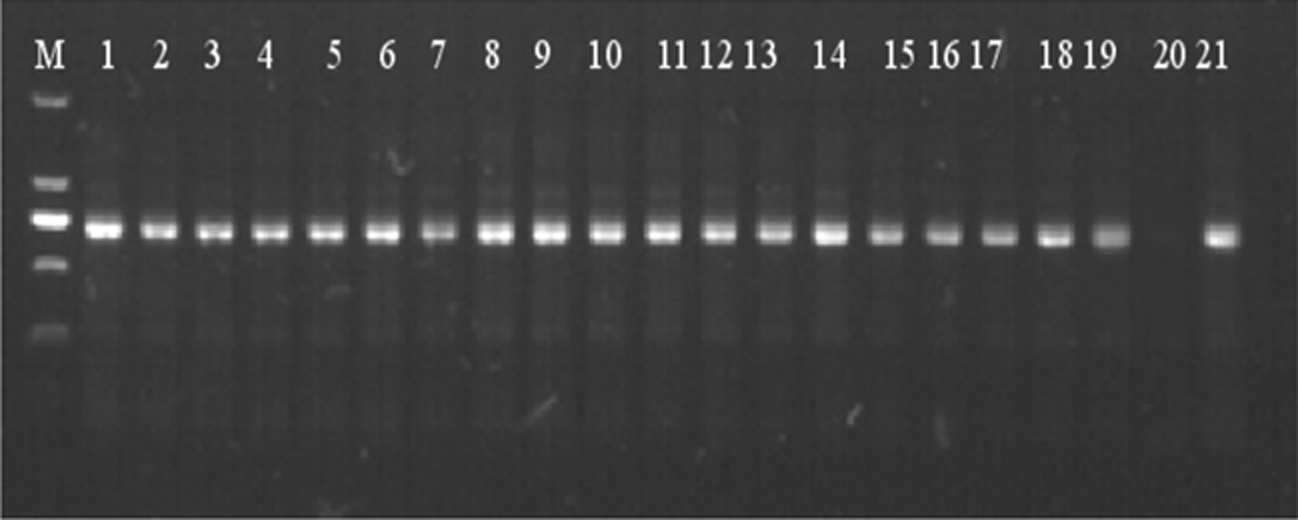

Supplement: S10 Fig — M: DL2000 Marker; 1–21: Nulli-tetrasomic lines respectively lacking 1A, 1B, 1D, 2D, 2A, 2B, 3D, 3B, 3A, 4B, 4D, 4A, 5D, 5B, 5A, 6D, 6B, 6A, 7A, 7D, 7B chromosome. TaPR1 is located on 7D. (TIF) [file pgen.1008713.s010.tif]

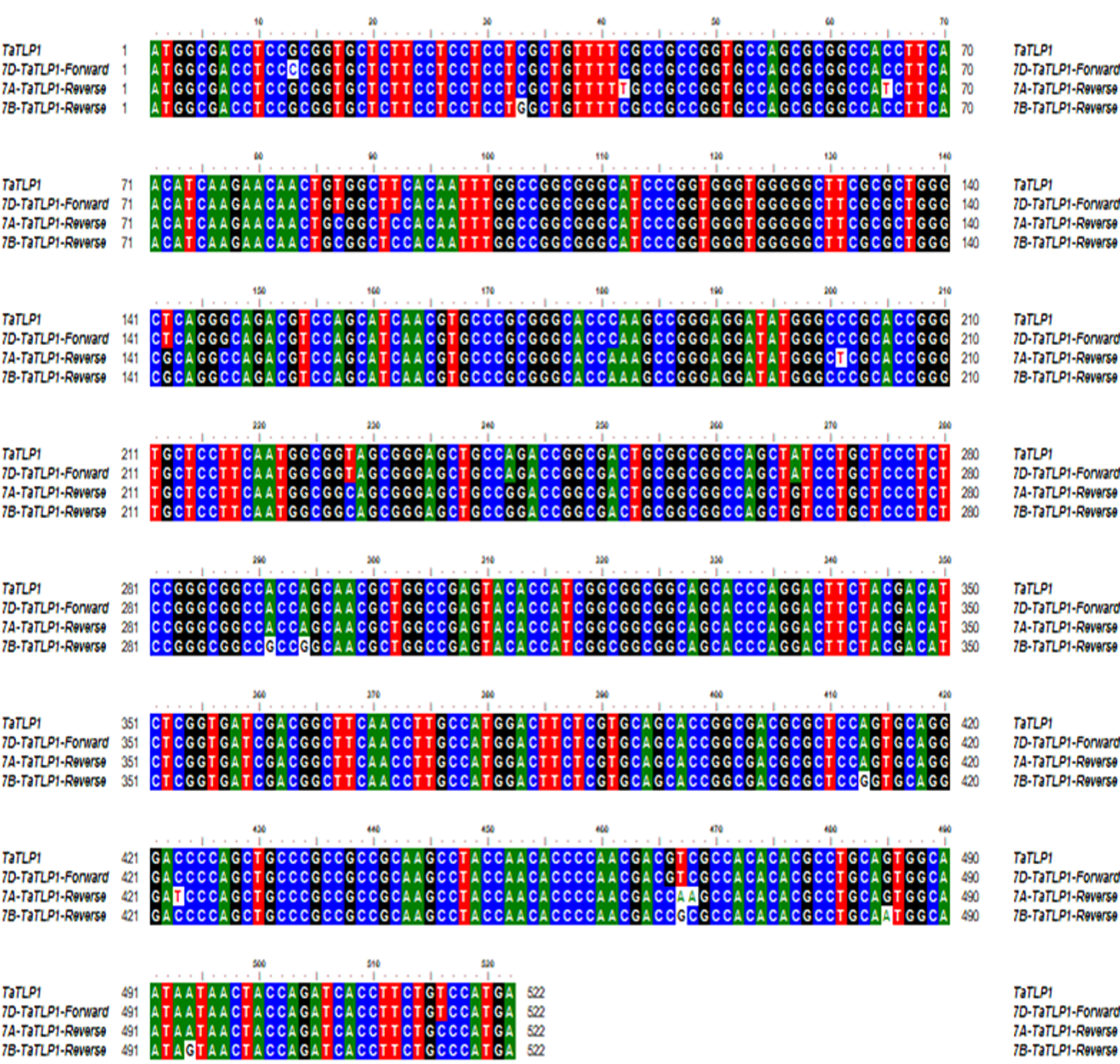

Supplement: S11 Fig — (TIF) [file pgen.1008713.s011.tif]

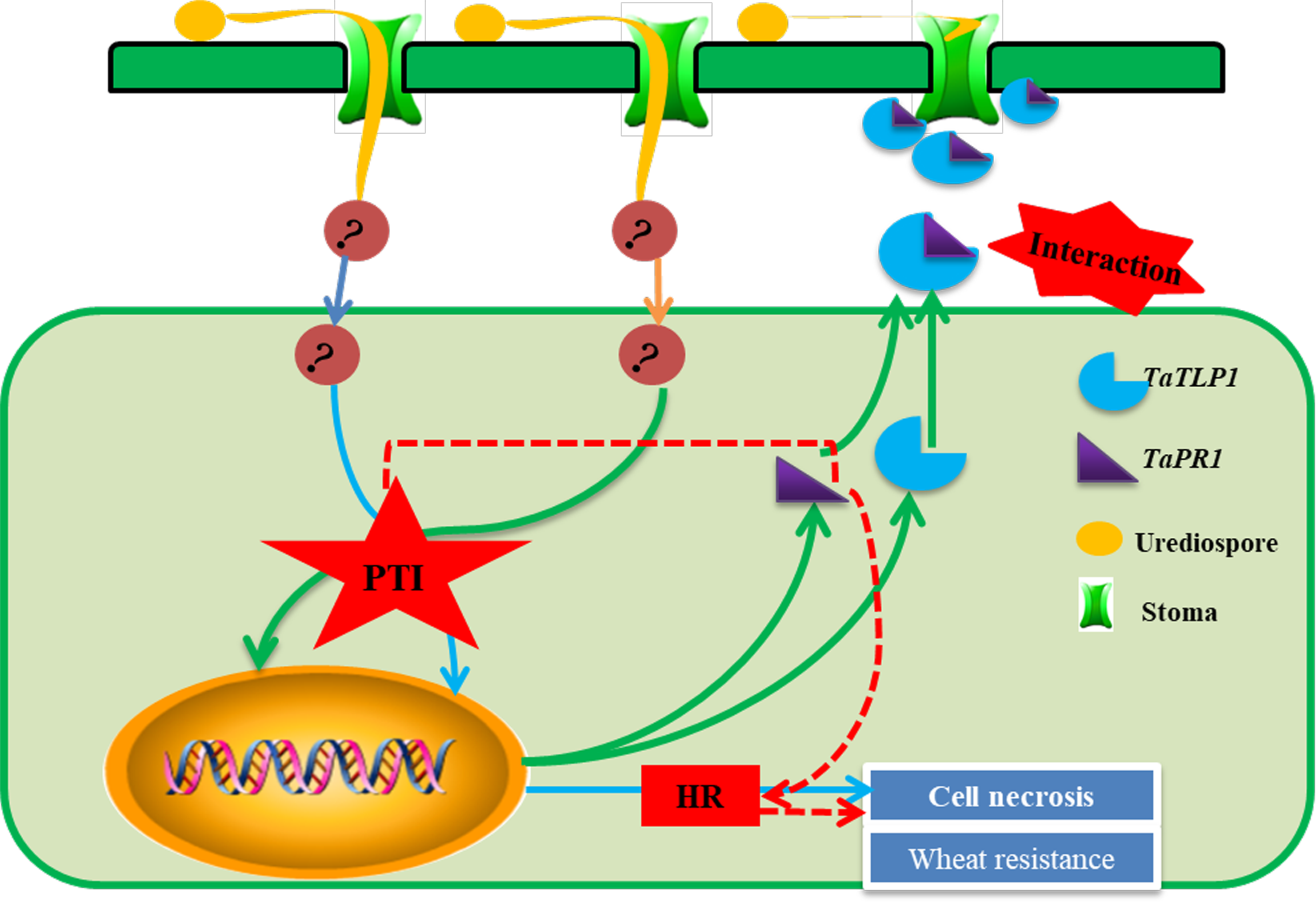

Supplement: S12 Fig — Upon Pt infection wheat, a receptor localized in plant plasma membrane recognizes an unknown molecule of Pt, activating cellular signaling PTI and producing TaTLP1 and TaPR1. TaTLP1 could interact with TaPR1 and protect the wheat against fungal action, and regulated ROS generation in the interaction between wheat and Pt. (TIF) [file pgen.1008713.s012.tif]
