# Supplementary material for: TaTLP1 interacts with TaPR1 to contribute to wheat defense responses to leaf rust fungus
Source: PLoS Genet. 2020 Jul 13;16(7):e1008713. doi: 10.1371/journal.pgen.1008713 (PMC7357741; doi:10.1371/journal.pgen.1008713)
Supplement: S1 Table — (DOCX) [file pgen.1008713.s013.docx]

| Table 1 Proteins identified in MS analyses after GFP-Trap | | | | |
| --- | --- | --- | --- | --- |
| Protein name | Gene ID | Annotation | Gene score | Counts |
| Pathogenesis-related protein 1 | LOC107768378 | Cysteine-rich secretory proteins, antigen 5, and pathogenesis-related 1 proteins, domain of pathogenesis-related protein 1 (PR-1) family proteins. | 521 | 20 |
| Endochitinase B | LOC107826794 | Chitinase class I, carbohydrate binding site [chemical binding], Hevein or Type 1 chitin binding domain subfamily co-occuring with family 19 glycosyl hydrolases or barwin domains. | 1008 | 30 |
| 14-3-3-like protein A | LOC107794654 | Dimer interface, peptide binding site [polypeptide binding]. | 707 | 32 |
|  |  |  |  |  |
| Osmotin | LOC107787819 | Lycoside hydrolase family 64 (beta-1,3-glucanases which produce specific pentasaccharide oligomers) and thaumatin-like proteins. | 415 | 31 |
|  |  |  |  |  |
| Osmotin-like | LOC107794479 | Lycoside hydrolase family 64 (beta-1, 3-glucanases which produce specific pentasaccharide oligomers) and thaumatin-like proteins. | 362 | 15 |
| Non-specific lipid-transfer protein | LOC107807546 | Non-specific lipid-transfer protein type 1 (nsLTP1) subfamily; Plant nsLTPs are small, soluble proteins that facilitate the transfer of fatty acids, phospholipids, glycolipids, and steroids between membranes. In addition to lipid transport and assembly. | 266 | 15 |
| Thaumatin-like protein | LOC107814286 | Lycoside hydrolase family 64 (beta-1,3-glucanases which produce specific pentasaccharide oligomers) and thaumatin-like proteins. | 208 | 22 |
