# Supplementary material for: TaTLP1 interacts with TaPR1 to contribute to wheat defense responses to leaf rust fungus
Source: PLoS Genet. 2020 Jul 13;16(7):e1008713. doi: 10.1371/journal.pgen.1008713 (PMC7357741; doi:10.1371/journal.pgen.1008713)
Supplement: S2 Table — (DOCX) [file pgen.1008713.s014.docx]

Table 2 Antifungal activity of TaTLP1-TaPR1

| Group | Number of spores | Number of germinated spores | Number of ungerminated spores | Germination rate (%) | Hyphae length (μm) |
| --- | --- | --- | --- | --- | --- |
| Sterile water | 113±5.49a | 110.59±0.97a | 2.41±0.51a | 97.86±0.17a | 1925.76±4.71a |
| Elution buffer | 149.4±4.89a | 145±4.41ab | 4.40±0.49a | 96.99±0.24a | 1885.493±7.979ab |
| pGEX-6P-3 vector | 138.8±6.86b | 118.8±0.28a | 31.5±0.24b | 78.3±0.41b | 1705.272±9.02ab |
| TaPR1 pure protein | 95±3.2b | 14.36±2.17bc | 80.64±0.73c | 15.12±2.09c | 767.18±4.52c |
| TaTLP1 pure protein | 109.1±7.5b | 11.70±1.55d | 97.4±0.65cd | 10.72±2.58d | 653.403±5.01d |
| TaPR1-TaTLP1 pure protein | 95±3.8b | 10.3±0.49cd | 84.7±1.39cd | 10.84±4.83d | 580.457±6.583d |
