# Supplementary material for: TaTLP1 interacts with TaPR1 to contribute to wheat defense responses to leaf rust fungus
Source: PLoS Genet. 2020 Jul 13;16(7):e1008713. doi: 10.1371/journal.pgen.1008713 (PMC7357741; doi:10.1371/journal.pgen.1008713)
Supplement: S3 Table — (DOCX) [file pgen.1008713.s015.docx]

Table 3 List of primers used in this study

| Primer name | Primer sequence |
| --- | --- |
| △spTaTLP1-pENTR-F | *CACC* GCCACCTTCTACATCAAGAACAACTGC |
| △spTaTLP1-pENTR-R | TCATGGACAGAAGGTGATCTGGTAG |
| △spTaPR1-pENTR-F | *CACC* CAGAACTCGCCTCAGGACTACCTCTCAC |
| △spTaPR1-pENTR-R | GTATGGTTTCTGTCCAATGATATTCCCG |
| TaTLP1-pENTR-F | *CACC* ATGGCGACCTCCGCGGTGCTCTTC |
| TaPR1-pENTR-R | TCATGGACAGAAGGTGATCTGGTAG |
| TaPR1-pENTR-F | *CACC* ATGCAGACGCCCAAGCTAGCCATCTTG |
| TaPR1-pENTR-R | GTATGGTTTCTGTCCAATGATATTCCCG |
| TaTLP1-Overlap-F | *GAATTC* GCCACCTTCTACATCAAGAACAACTGC |
| Overlap-TaTLP1-F1 | TGGACTTCTCGGCAGGCACC |
| Overlap-TaTLP1-R1 | GGTGCCTGCCGAGAAGTCCA |
| Overlap-TaTLP1-F2 | CCAGGCAAGGGACCCCAGC |
| Overlap-TaTLP1-R2 | GCTGGGGTCCCTTGCCTGG |
| Overlap-TaTLP1-F3 | CGCACCGCCGCCGCAAGCC |
| Overlap-TaTLP1-R3 | GGCTTGCGGCGGCGGTGCG |
| Overlap-TaTLP1-F4 | ACGCCGCAAATGGCAACA |
| Overlap-TaTLP1-R4 | TGTTGCCATTTGCGGCGT |
| TaTLP1-Overlap-R | GGATCC TCATGGTGCGAAGGTGATCTGGTAG |
| qTaTaPR1-F | CAATAACCTCGGCGTCTTCATC |
| qTaTaPR1-R | ATTTACTCGCTCGGTCCCTC |
| TaPR1-VIGS-F1 | *TTAATTAA* ATGCAGACGCCCAAGCTA |
| TaPR1-VIGS-R1 | *GCGGCCGC* CATCTTCTGGGGGTCGGCCGGCG |
| TaPR1-VIGS-F2 | *TTAATTAA*CGGACTGGAAGGCGGCGGA |
| TaPR1-VIGS-R2 | *GCGGCCGC* ATCATTGGACAGAAACCATACTAA |
| TaPR1-45 siteN-F | *GAATTC* GCGGTGACCTGGAGCACGAAGC |
| TaPR1-45 siteN-R | *GGATCC* TGGTTTCTGTCCAATGATATTCCCG |
| TaPR1-64 siteN-F | *GAATTC* ATCAACGACTGCAAGCTCCAGC |
| TaPR1-64 siteN-R | *GGATCC* TTAGTATGGTTTCTGTCCAATGATA |
| TaPR1 siteC-F | *GAATTC* CAGAACTCGCCTCAGGACTACCTCTCAC |
| TaPR1-113 siteC-R | *GGATCC* GCAGGTGTTGGAGCCGTAG |
| TaPR1-128 siteC-R | *GGATCC* GCCACACCACCTGCGTGTAGTGC |
| TaPR1-143 siteC-R | *GGATCC* GTTATTGTTGCAGACGACGCGGG |
| SCS265-F | GGATAAGCAGAGCAGAG |
| SCS265-R | GGCGGATAAGTGGGTTATGG |
| SCS253-F | GCTGGTTCCACAAAGCAAA |
| SCS253-R | GGCTGGTTCCTTAGATAGGTG |
| TaTLP1-VIGS2-F | *TTAATTAA* ATGGCGACCTCCGCGGTGCTC |
| TaTLP1-VIGS2-R | GCGGCCGC CTACCGCCATTGAAGGAG |
| qTaTLP1-F  qTaTLP1-R | GGGATCCATGGCGACCTCCGCGGTGCTC  CCAAGCTTTCATGGACAGAAGGTGATCTGGTC |
| TaPR1 Biat-F | *GAATTC* CAGAACTCGCCTCAGGACTACCTCTCAC |
| TaPR1 Biat-R | *GGATCC* GTATGGTTTCTGTCCAATGATATTCCCG |
| TaTLP1 Biat-F | GAATTC GCCACCTTCTACATCAAGAACAACTGC |
| TaTLP1 Biat-R | GGATCC TCATGGACAGAAGGTGATCTGGTAG |
| q-GAPDH-F | TGCCTTGCTCGTCTTGCTAA |
| q-GAPDH-R | CTTGATGGAAGGACCATCAAC |
| q-TaSOD-F | GGCTCTCCAAGGTCGTGT |
| q-TaSOD-R | GGGTTGCCGTTGTTGTAG |
| q-TaCAT-F | GCCTGTGTTTTTTATCCGAGA |
| q-TaCAT-R | CAGGTGCCTCCAACAGTAACA |
