# Supplementary material for: TaTLP1 interacts with TaPR1 to contribute to wheat defense responses to leaf rust fungus
Source: PLoS Genet. 2020 Jul 13;16(7):e1008713. doi: 10.1371/journal.pgen.1008713 (PMC7357741; doi:10.1371/journal.pgen.1008713)
Supplement: Attachment — Submitted filename: Comments from reviewer Plos Genetics.docx [file pgen.1008713.s016.docx]

In the manuscript the authors attempt to show that TaTLP1 and TaPR1 interact with one another and contribute to resistance against *Puccinia triticina.* They perform pull-down experiments to identifyTaPR1, and the interaction between TaTLP1 and TaPR1 was confirmed by yeast two-hybrid techniques, bimolecular fluorescence complementation (BiFC), and co-immunoprecipitation. They also used TaTLP1-TaPR1 co-silenced plants along with single knock down plants to show that there is an increase in susceptibility. The experiments indicate that the two proteins interact and these proteins contribute to resistance. But the major concern is that the experiments do not conclusively prove that this interaction contributes to resistance. The data can be interpreted as the proteins having overlapping functions or roles in resistance.

**Some specific points:**

The grammar and syntax throughout the manuscript need to be corrected. Some sentences are difficult to decipher.

**Abstrtact:**

The last line of the abstract can be re-written such that it reflects the findings about TaTLP1 and TaPR1 only instead of including all PR proteins in general.

**Author Summary:**

The first few lines of the summary can be shortened or deleted. The paragraph can begin with and discuss Thaumatins or TLP proteins and their role in general.

Line 38: The words “Defense responses” may be more suitable than “disease-resistance responses”,

**Introduction:**

Abbreviations and names should be explained at first. E.g. line 146: PHNT, the term Thatcher should also be explained. These terms and other abbreviated terms should be clearly described in the materials and methods section also.

**Results:**

Line 151-153: The fact that their expression profiles are similar under compatible and incompatible interaction does not show that they interact with each other, at this point. The sentence should be changed. The Y2H, BiFC co-localization experiments etc. subsequently described prove they do interact. So this fact can be claimed at a later point.

Figure 1: The gel photo is not distinct enough to see the indicated protein band(s). Moreover extra protein bands are visible in the -TaTLP1 at ca 30kDa, 25 kDa etc. Similarly extra protein bands are visible in the +TaTLP1 ca 45kDa, 50kDa etc.

Line 236: The experiments prove that these two proteins interact and that that the 52 amino acid conserved region of the TaPR1 is sufficient to bind the TaTLP1 but these do not indicate that this is “sufficient for defense to leaf rust”

Moreover the 52 amino acid truncated protein having only the conserved region may not bond with the same strength as that required for binding in vivo under diseased condition. So perhaps an experiment to show the strength of bonding between these two proteins (truncated TaPR1 and TaTLP1) in vitro or in transient expression experiments can shed some light. A preliminary prediction of Protein-Protein Interaction strength can be made using software.

Under diseased condition where there are several proteins which are differentially expressed, the situation can alter the affinity of the binding between these two proteins. This fact can be discussed in the discussion part.

Line 283-285: “antifungal activity of both TaTLP1-TaPR1 and TaTLP1 or TaPR1 alone significantly increased compared with control” So the inference is that these protein alone or in combination have antifungal activity. But the heading (line 269-270) does not agree with this. The heading seems to say that the protein in combination has more antifungal activity than the individual proteins. If that is so, then the sentence “that TaTLP1-TaPR1 could protect wheat by...” should also be changed to “the proteins alone or in combination can protect wheat by.....”.

**Discussion**

Much of the discussion is repetition of the results (e.g. Lines: 403-407, 427-460). Discussion should focus on the interpretation of the data with respect to prior findings and the significance of the findings.

Material and methods:

This section does not clearly state the host varieties used and the pathogen sources etc. The “materials” or organisms used and their sources should be mentioned.

**References that can be included:**

[Anand A](https://www.ncbi.nlm.nih.gov/pubmed/?term=Anand%20A%5BAuthor%5D&cauthor=true&cauthor_uid=12598580)^1^, [Zhou T](https://www.ncbi.nlm.nih.gov/pubmed/?term=Zhou%20T%5BAuthor%5D&cauthor=true&cauthor_uid=12598580), [Trick HN](https://www.ncbi.nlm.nih.gov/pubmed/?term=Trick%20HN%5BAuthor%5D&cauthor=true&cauthor_uid=12598580), [Gill BS](https://www.ncbi.nlm.nih.gov/pubmed/?term=Gill%20BS%5BAuthor%5D&cauthor=true&cauthor_uid=12598580), [Bockus WW](https://www.ncbi.nlm.nih.gov/pubmed/?term=Bockus%20WW%5BAuthor%5D&cauthor=true&cauthor_uid=12598580), [Muthukrishnan S](https://www.ncbi.nlm.nih.gov/pubmed/?term=Muthukrishnan%20S%5BAuthor%5D&cauthor=true&cauthor_uid=12598580). Greenhouse and field testing of transgenic wheat plants stably expressing genes for thaumatin-like protein, chitinase and glucanase against Fusarium graminearum. [*J Exp Bot.*](https://www.ncbi.nlm.nih.gov/pubmed/12598580) 2003, 54(384):1101-11.

Chowdhury S, Basu A, Kundu S. Overexpression of a New Osmotin-Like Protein Gene (*SindOLP*) Confers Tolerance against Biotic and Abiotic Stresses in Sesame. *Front Plant Sci*. 2017;8:410. Published 2017 Mar 28. doi:10.3389/fpls.2017.00410

de Jesus-Pires C, Ferreira-Neto JRC, Pacifico Bezerra-Neto J, Kido EA, de Oliveira Silva RL, Pandolfi V, Wanderley-Nogueira AC, Binneck E, da Costa AF, Pio-Ribeiro G, Pereira-Andrade G, Sittolin IM, Freire-Filho F, Benko-Iseppon AM. [Plant Thaumatin-like Proteins: Function, Evolution and Biotechnological Applications.](https://www.ncbi.nlm.nih.gov/pubmed/30887921) *Curr Protein Pept Sci.* 2019 Mar 18. doi: 10.2174/1389203720666190318164905.

Faillace GR, Turchetto-Zolet AC, Guzman FL, de Oliveira-Busatto LA, Bodanese-Zanettini MH.[Genome-wide analysis and evolution of plant thaumatin-like proteins: a focus on the origin and diversification of osmotins.](https://www.ncbi.nlm.nih.gov/pubmed/31030277) *Mol Genet Genomics.* 2019 Apr 27. doi: 10.1007/s00438-019-01554-y.

[Mackintosh CA](https://www.ncbi.nlm.nih.gov/pubmed/?term=Mackintosh%20CA%5BAuthor%5D&cauthor=true&cauthor_uid=17103001)^1^, [Lewis J](https://www.ncbi.nlm.nih.gov/pubmed/?term=Lewis%20J%5BAuthor%5D&cauthor=true&cauthor_uid=17103001), [Radmer LE](https://www.ncbi.nlm.nih.gov/pubmed/?term=Radmer%20LE%5BAuthor%5D&cauthor=true&cauthor_uid=17103001), [Shin S](https://www.ncbi.nlm.nih.gov/pubmed/?term=Shin%20S%5BAuthor%5D&cauthor=true&cauthor_uid=17103001), [Heinen SJ](https://www.ncbi.nlm.nih.gov/pubmed/?term=Heinen%20SJ%5BAuthor%5D&cauthor=true&cauthor_uid=17103001), [Smith LA](https://www.ncbi.nlm.nih.gov/pubmed/?term=Smith%20LA%5BAuthor%5D&cauthor=true&cauthor_uid=17103001), [Wyckoff MN](https://www.ncbi.nlm.nih.gov/pubmed/?term=Wyckoff%20MN%5BAuthor%5D&cauthor=true&cauthor_uid=17103001), [Dill-Macky R](https://www.ncbi.nlm.nih.gov/pubmed/?term=Dill-Macky%20R%5BAuthor%5D&cauthor=true&cauthor_uid=17103001), [Evans CK](https://www.ncbi.nlm.nih.gov/pubmed/?term=Evans%20CK%5BAuthor%5D&cauthor=true&cauthor_uid=17103001), [Kravchenko S](https://www.ncbi.nlm.nih.gov/pubmed/?term=Kravchenko%20S%5BAuthor%5D&cauthor=true&cauthor_uid=17103001), [Baldridge GD](https://www.ncbi.nlm.nih.gov/pubmed/?term=Baldridge%20GD%5BAuthor%5D&cauthor=true&cauthor_uid=17103001), [Zeyen RJ](https://www.ncbi.nlm.nih.gov/pubmed/?term=Zeyen%20RJ%5BAuthor%5D&cauthor=true&cauthor_uid=17103001), [Muehlbauer GJ](https://www.ncbi.nlm.nih.gov/pubmed/?term=Muehlbauer%20GJ%5BAuthor%5D&cauthor=true&cauthor_uid=17103001). Overexpression of defense response genes in transgenic wheat enhances resistance to Fusarium head blight. [*Plant Cell Rep*.](https://www.ncbi.nlm.nih.gov/pubmed/17103001) 2007 Apr;26(4):479-88. Epub 2006 Nov 11.

Van der Maelen E, Rezaei MN, Struyf N, Proost P, Verstrepen KJ, Courtin CM. [Identification of a Wheat Thaumatin-like Protein That Inhibits *Saccharomyces cerevisiae*.](https://www.ncbi.nlm.nih.gov/pubmed/31487168)

*J Agric Food Chem.* 2019 Sep 18;67(37):10423-10431. doi: 10.1021/acs.jafc.9b03432. Epub 2019 Sep 5.

Xing L.-P., Wang H.-Z., Jiang Z.-N., Ni J.-L., Cao A.-Z., Yu L., Chen P.-D.

Transformation of Wheat Thaumatin-Like Protein Gene and Analysis of Reactions to Powdery Mildew and Fusarium Head Blight in Transgenic Plants

(2008)  *Acta Agronomica Sinica*,  34  (3) , pp. 349-354.
