# Supplementary material for: TaTLP1 interacts with TaPR1 to contribute to wheat defense responses to leaf rust fungus
Source: PLoS Genet. 2020 Jul 13;16(7):e1008713. doi: 10.1371/journal.pgen.1008713 (PMC7357741; doi:10.1371/journal.pgen.1008713)
Supplement: Attachment — Submitted filename: dongmeiwang--suggestions.doc [file pgen.1008713.s017.doc]

**The comments on the manuscript with the title 'TaTLP1 interacts with TaPR1 to contribute to wheat defense responses to leaf rust fungus' (PGENETICS-D-19-01570)**

In this work, the authors studyed the functions of *TaLTP* and *TaPR1* ,and the interection between TaLTP and TaPR1 .Generally speaking, the work is well done , so the results are expectable.

According to the MS, I have some suggestions as following:

1.The English language is required to be improved by any reputable English language editing service. Some mistakes should be corrected through your manuscript such as TaTLP1 and TaPR1(*TaTLP1* and *TaPR1*) from line 321 of page 12.

2.The descriptions of "Backgroud" section and "Discussion" are inadequate without the latest research progress, especially since 2018.

3.The title should be clearer,please see line 235 of page 9.

4. Please unify the statement:P*uccinia triticina,*leaf rust,and leaf rust fungus.

5.The line 282 of page 11:Microscopic observation showed that the germination of urediniospores and the growth of hyphae were significantly restricted with pure proteins,From the Fig7, it is not a hyphae but a germtube.

6.Please mark the bar of fig9 and fig10.

7.See line 369 of page 14:The areas of cell death and H_2_O_2_ accumulation was significantly lower at 120 hpi in TaTLP1/TaPR1-knockdown plants than that observed in BSMV:γ, TaTLP1 or TaPR1 alone inoculated leaves,please give the statistical result of the areas of cell death. Analyze the reasons in detail.

Dongmei Wang
