# Supplementary material for: TaTLP1 interacts with TaPR1 to contribute to wheat defense responses to leaf rust fungus
Source: PLoS Genet. 2020 Jul 13;16(7):e1008713. doi: 10.1371/journal.pgen.1008713 (PMC7357741; doi:10.1371/journal.pgen.1008713)
Supplement: Attachment — Submitted filename: Response letter.docx [file pgen.1008713.s018.docx]

Dear Editor and Reviewers,

We would like to thank you for the constructive inputs on our manuscript. We have revised the manuscript based on your comments. Your comments are all valuable and very helpful for revising and improving our paper, as well as putting into perspective the significance of our research. Now, further improvements have been provided to ensure the quality of the manuscript which we hope will be met with approval. Revised portions are marked in red in the paper. The main corrections in the paper and the responds to the reviewer’s comments are as follows:

**Response to the editor’s comments:**

In particular, the discussion should be re-written to place results in context. Furthermore, the work presented here needs more careful examination and additional experimentation that explain why interaction of PR1 and TLP in the apoplast is important for wheat defence against a biotroph fungus that forms intimate connection with the host through formation of haustoria inside the cell.

**Response:** We have re-phased the discussion and added the corresponding information based on the results. Indeed, leaf rust forms intimate connection with the host through formation of haustoria. As we know, the haustoria only bind the cell membrane tightly and do not penetrate the cell membrane, so the interaction between host and fungus happens in the space between the cell membrane and cell wall, but not inside the cell. We added the corresponding information in the discussion section according to the reference as following:

**Reference:** Panstruga, R., Dodds, P. N. 2009. Terrific protein traffic: the mystery of effector protein delivery by filamentous plant pathogens. Science, 324(5928):748-50.

Haustoria emerge from extracellular pathogen infection structures (such as spores or hyphae) and enter plant cells through the infection peg by invaginating the host plasma membrane. Haustoria are surrounded by the pathogen plasmalemma and cell wall as well as the modified host plasma membrane (extrahaustorial membrane). These membranes enclose the extrahaustorial matrix, an interface zone that is sealed by the neck band.

**Reviewer #1:**

**1.Comment:** But the major concern is that the experiments do not conclusively prove that this interaction contributes to resistance. The data can be interpreted as the proteins having overlapping functions or roles in resistance.

**Response:** In our experiment, in order to prove that this interaction contributes to resistance, we listed three results as following: 1. MG132, a proteasome inhibitor, was used to examine and compare the stability and accumulation of TaTLP1 to test whether it was regulated by TaPR1, and the results suggest that TaPR1 stabilizes TaTLP1 by preventing its degradation mediated by the 26S proteasome. 2. Antifungal activity of TaTLP1-TaPR1 is higher when compared to either TaTLP1 or TaPR1 alone *in vitro*. 3. Co-silencing of *TaTLP1* and *TaPR1* using VIGS significantly enhances wheat susceptibility compared with single gene silencing after inoculation with *Pt*. The data show that both proteins additively contribute to antifungal activity and resistance against the pathogen *Puccinia triticina.* Of course, in the future, we will try to reveal the biochemical parameters that would explain how the complex between TaTLP1 and TaPR1 enhances their anti-microbial activities.

**2. Comment:** Some specific points:

(1) The grammar and syntax throughout the manuscript need to be corrected. Some sentences are difficult to decipher.

**Response:** We checked whole the manuscript and corrected the mistakes.

(2) Abstract: The last line of the abstract can be re-written such that it reflects the findings about TaTLP1 and TaPR1 only instead of including all PR proteins in general.

**Response:** We revised the last sentence according to reviewer’s suggestion.

(3) Author Summary: The first few lines of the summary can be shortened or deleted. The paragraph can begin with and discuss Thaumatins or TLP proteins and their role in general.

**Response:** The first few lines of the summary was deleted according to reviewer’s suggestion.

(4) Line 38: The words “Defense responses” may be more suitable than “disease-resistance responses”

**Response:** Thank you for this suggestion, we have changed our phrasing.

(5) Introduction: Abbreviations and names should be explained at first. E.g. line 146: PHNT, the term Thatcher should also be explained. These terms and other abbreviated terms should be clearly described in the materials and methods section also.

**Response:** We revised it according to the reviewer’s suggestion.

(6) Results: Line 151-153: The fact that their expression profiles are similar under compatible and incompatible interaction does not show that they interact with each other, at this point. The sentence should be changed. The Y2H, BiFC co-localization experiments etc. subsequently described prove they do interact. So this fact can be claimed at a later point.

**Response:** We have re-phased this section.

(7) Figure 1: The gel photo is not distinct enough to see the indicated protein band(s). Moreover extra protein bands are visible in the -TaTLP1 at ca 30kDa, 25 kDa etc. Similarly extra protein bands are visible in the +TaTLP1 ca 45kDa, 50kDa etc.

**Response:** We used a new image to show the results according to the reviewer’s suggestion.

(8) Line 236: The experiments prove that these two proteins interact and that that the 52 amino acid conserved region of the TaPR1 is sufficient to bind the TaTLP1 but these do not indicate that this is “sufficient for defense to leaf rust”

**Response:** Right idea, we deleted “sufficient for defense to leaf rust”.

(9) Moreover the 52 amino acid truncated protein having only the conserved region may not bond with the same strength as that required for binding in vivo under diseased condition. So perhaps an experiment to show the strength of bonding between these two proteins (truncated TaPR1 and TaTLP1) in vitro or in transient expression experiments can shed some light. A preliminary prediction of Protein-Protein Interaction strength can be made using software.

**Response:** As you suggest we used the truncated TaPR1 and TaTLP1 to show the strength of bonding between these two proteins *in vitro*. In addition, a preliminary prediction of protein-protein interaction strength was made using software and listed as a supplementary information.

(10) Under diseased condition where there are several proteins which are differentially expressed, the situation can alter the affinity of the binding between these two proteins. This fact can be discussed in the discussion part.

**Response:** As you suggest we discussed this idea in the discussion section.

(11) Line 283-285: “antifungal activity of both TaTLP1-TaPR1 and TaTLP1 or TaPR1 alone significantly increased compared with control” So the inference is that these proteins alone or in combination have antifungal activity. But the heading (line 269-270) does not agree with this. The heading seems to say that the protein in combination has more antifungal activity than the individual proteins. If that is so, then the sentence “that TaTLP1-TaPR1 could protect wheat by...” should also be changed to “the proteins alone or in combination can protect wheat by.....”.

**Response:** We have re-phased the sentence according to the reviewer’s suggestion.

(12) Discussion

Much of the discussion is repetition of the results (e.g. Lines: 403-407, 427-460). Discussion should focus on the interpretation of the data with respect to prior findings and the significance of the findings.

**Response:** We have re-phased the discussion according to the reviewer’s suggestion.

(13) Material and methods: This section does not clearly state the host varieties used and the pathogen sources etc. The “materials” or organisms used and their sources should be mentioned.

**Response:** The information of host varieties used and the pathogen sources etc. have been added to the Material and Methods section.

**Reviewer #2:**

**1. Comment:** There were several strong candidates in addition to PR1 as potential interactions of TLP. Some of these proteins have reported function in plant immunity and they had higher count than PR1 (Table1 1). Authors should examine interaction of these targets by Y2H and Co-IP?

**Response:** Yes, there were several strong candidates in addition to TaPR1 as potential interactions of TaTLP1. Because we have better research background about TaPR1, we paid much attention to PR1-like protein co-purified with TaTLP1 by GFP trap. We checked the results separated by SDS-PAGE, an 18-kD protein band was co-purified with TaTLP1, whereas no additional band appeared in the control assays with extracts alone (Fig 1). We will examine interaction of other targets by Y2H and Co-IP in the future.

**2. Comment:** Lines 195-196 and Fig 4B. Apoplast localization of PR1-TLP needs to be verified by co- localization of and apoplast marker.

**Response:** We changed Fig 4B to show apoplast localization by plasmolysis treatment.

**3. Comment:** Lines 216-234 and Fig 5B. Authors claim that TaPR1 stabilizes the accumulation of TaTLP protein. However the amount of protein in lane 2 is visibly less than lane 1 in fig 5 B. it is not clear why authors used MG132 when there is no evidence that PR1 and TLP are targeted to the proteasome pathway. Also myc control was used by there is no Myc antibody labelling. the order of lower panel Ha, GFP labelling should be revered so it correspond to the same order of the upper panel proteins .

**Response:** We are sorry there are some mistakes. We have revised them and changed the order of the labelling.

**4. Comment:** L296-287: VIGS of PR1 and TLP1

A- why level of expression for PR1 and TLP1 are higher at 48 hrs compare to 24 hrs and goes down again at later time point. it is expected that as time progress level of expression of target genes should reduce or at least stay the same since the virus multiply and spreads. To knock down the expression of both PR1 and TLP1 a VIGS construct with fragments for PR1 and TLP1 should be used instead of mixing the two.

**Response:** According to reviewer’s question, we discussed it in manuscript. VIGS is a transient expression system, normally 10 days after BSMV inoculation, leaf rust urediospores were applied with a brush to the surface of the leaves of wheat. We compare the expression level of *TaPR1* and *TaTLP1* to BSMV:γ as control to identify the silencing efficiency in Fig. 8. Similar research on stripe rust show similar results**,** such as a paper entitled **“**TaCIPK10 interacts with and phosphorylates TaNH2 to activate wheat defense responses to stripe rust”.

B- Fig. 8 needs proper labelling and caption need proper description to show which wheat cv was used. HR in the positive control leaf is not visible in the image. Pathogen growth seems to be the same in the PR1/TLP1 combination compared to PR1 or TLP1 alone as also seen in Fig 9.

**Response:** Thank you for this suggestion, we have changed the labelling to show wheat cv. The pathogen growth including the length of the hyphae, the area of infection, and the number of mycelial branches of *Pt* in wheat leaves was shown by the [column](http://cn.bing.com/dict/search?q=column&FORM=BDVSP6&mkt=zh-cn) [chart](http://cn.bing.com/dict/search?q=chart&FORM=BDVSP6&mkt=zh-cn).

C- Fig 10: H_2_O_2_ level needs to be quantified. it is hard to just from the image conclude that H_2_O_2_ level is reduced. Authors need to describe why they used SOD and CAT and how the change in tier expression correlate with the level of H_2_O_2_.

**Response:** H_2_O_2_ levels have been quantified, and we have described the reason why *SOD* and *CAT* correlate with the level of H_2_O_2_ was use.

**5. Comment:** Finally, manuscript needs to be edited extensively to improve the writing. Introduction should include a background about the leaf rust, genetic of resistance and what is known about the pathogen interaction with wheat.

**Response:** We have re-phased the Introduction according to the reviewer’s suggestion.

**6. Comment:** results are not descriptive enough in some places and use of abbreviation should be described when first appears in the text.

**Response:** We checked and revised results section and expanded the abbreviation when it first appears in the text.

**7. Comment:** Discussion is mainly repeating the results and it does not discuss some of the main conclusion (e.g. stability of TLP1)
**Response:** We have re-phased the discussion according to the reviewer’s suggestion.

**Reviewer #3:**

1. **Comment:**

Line 23: In vitro, the…

Line 65: expand BcIEB1 and B. cinereal

Line 67: expand PevD1

Line 79: expand SsCP1

Line 80: expand GST

**Response:** We expanded the abbreviation when it first appears in the text.

**2.Comment:** Page 4; Instead of talking about PTI and ETI please explain about race specific and APR resistance in wheat rust resistance. Also provide details that TLP1 and PR1 are found to be important in both these types (ref some papers such as Sr21, Sr13, Lr34 etc papers). Also point that for the first time we report the interaction of these two molecules in wheat if its not been reported earlier.

**Response:** Thanks for your good suggestion, we have re-phased it.

**3. Comment:** Line 146: Pt race PHNT

Page 10: Please indicate CAPE1 what is the amino acid region

Page 13: Please indicate that silencing of both TLP and PR produce slightly larger pustules as compared to the control

Line 408: CAPE1 is not required

Page 26: Please indicate the contribution of Xiong Du and Daqun Liu

**Response:** According to reviewer’s suggestion, we revised these sections.

**Reviewer #4:**

**1.Comment:** The English language is required to be improved by any reputable English language editing service. Some mistakes should be corrected through your manuscript such as TaTLP1 and TaPR1(TaTLP1 and TaPR1) from line 321 of page 12.

**Response:** We checked whole the manuscript, and the grammar and syntax throughout the manuscript was improved by one English language editing service.

**2. Comment:** The descriptions of "Background" section and "Discussion" are inadequate without the latest research progress, especially since 2018.

**Response:** We have re-phased the "Background" section and "Discussion" according to the reviewer’s suggestion.

**3. Comment:** The title should be clearer, please see line 235 of page 9.

**Response:** Yes, we changed the title.

**4. Comment:** Please unify the statement: *Puccinia triticina*, leaf rust, and leaf rust fungus.

**Response:** We checked and unified the statements.

**5. Comment:** The line 282 of page 11: Microscopic observation showed that the germination of urediniospores and the growth of hyphae were significantly restricted with pure proteins, From the Fig7, it is not a hypha but a germtube.

**Response:** Thanks for your comment. We revised it.

**6. Comment:** Please mark the bar of fig9 and fig10.

**Response:** We added the bar of Fig. 9 and Fig.10.

**7. Comment:** See line 369 of page 14: The areas of cell death and H_2_O_2_ accumulation was significantly lower at 120 hpi in TaTLP1/TaPR1-knockdown plants than that observed in BSMV:γ, TaTLP1 or TaPR1 alone inoculated leaves, please give the statistical result of the areas of cell death. Analyze the reasons in detail.

**Response:** We added the statistical result.

We deeply appreciate your consideration of our manuscript, and we look forward to receiving your answer. If you have any queries, please don’t hesitate to contact me at the address below.

Thank you and best regards.

Yours sincerely,

Haiyan Wang

Corresponding author:

Name: Haiyan Wang

E-mail: ndwanghaiyan@163.com
