# Supplementary material for: TaTLP1 interacts with TaPR1 to contribute to wheat defense responses to leaf rust fungus
Source: PLoS Genet. 2020 Jul 13;16(7):e1008713. doi: 10.1371/journal.pgen.1008713 (PMC7357741; doi:10.1371/journal.pgen.1008713)
Supplement: Attachment — Submitted filename: Comments from reviewer PGENETICS-D-19-01570R1.docx [file pgen.1008713.s019.docx]

The data are now better presented. The authors made the recommended corrections. There remain many syntax errors. The manuscript still needs to be rewritten in lucid English, starting from the abstract.
