# Supplementary material for: TaTLP1 interacts with TaPR1 to contribute to wheat defense responses to leaf rust fungus
Source: PLoS Genet. 2020 Jul 13;16(7):e1008713. doi: 10.1371/journal.pgen.1008713 (PMC7357741; doi:10.1371/journal.pgen.1008713)
Supplement: Attachment — Submitted filename: Response letter2.docx [file pgen.1008713.s020.docx]

Dear Editor and Reviewers,

We would like to thank you for the constructive inputs on our manuscript. We have revised the manuscript based on the comments. Please check below for the point-by-point responses.

**Response to the editor’s comments:**

Please address English Language writing in the paper and edit for clarity. Also, there are not sufficient details provided for the statistical analyses. What do the error bars represent? SE, SD? Not all the figures indicate the statistical test used. If ANOVA was used, what mean separation statistics were used after a significant F test? what N represents should also be included (individual plants, individual leaves, etc).

**Response:** Thanks for your suggestion. The grammar and syntax throughout the manuscript was improved. In addition, we have provided the details for the statistical analyses in figures and manuscript.

**Reviewer #1:**

**Comment:** The data are now better presented. The authors made the recommended corrections. There remain many syntax errors. The manuscript still needs to be rewritten in lucid English, starting from the abstract.

**Response:** We checked whole the manuscript, and the grammar and syntax throughout the manuscript was improved.

**Reviewer #3:** No comments

**Reviewer #4:**

**Comment:** Evidence from *in vivo* experiments is not sufficient

**Response:** In our experiment, in order to prove that this interaction contributes to resistance *in vivo*, we listed the results as following: 1. MG132, a proteasome inhibitor, was used to examine and compare the stability and accumulation of TaTLP1 to test whether it was regulated by TaPR1, and the results suggest that TaPR1 stabilizes TaTLP1 by preventing its degradation mediated by the 26S proteasome. 2. Co-silencing of *TaTLP1* and *TaPR1* using VIGS significantly enhances wheat susceptibility compared with single gene silencing after inoculation with *Pt*. Of course, in the future, we will try to reveal the biochemical parameters that would explain how the complex between TaTLP1 and TaPR1 enhances their anti-microbial activities, and an experiment to show the strength of bonding between these two proteins (truncated TaPR1 and TaTLP1) in transient expression experiments can shed some light.

We deeply appreciate your consideration of our manuscript, and we look forward to receiving your answer. If you have any queries, please don’t hesitate to contact me at the address below.

Thank you and best regards.

Yours sincerely,

Haiyan Wang

Corresponding author:

Name: Haiyan Wang

E-mail: ndwanghaiyan@163.com
